# The incidence of pedestrian distraction at urban intersections after implementation of a Streets Smarts campaign

**DOI:** 10.1186/s40621-015-0050-7

**Published:** 2015-08-15

**Authors:** Pina Violano, Linda Roney, Kirsten Bechtel

**Affiliations:** 1 Injury Prevention & Research Center, Yale-New Haven Hospital, New Haven, Connecticut USA; 2 Injury Free Coalition for Kids of New Haven, Yale-New Haven Children’s Hospital, 300 George St 4th Floor Room 443, New Haven, CT 06510 USA; 3 Fairfield University School of Nursing, 1058 N Benson Road, Fairfield, Connecticut 06824 USA; 4 Section of Pediatric Emergency Medicine, Yale School of Medicine, New Haven, USA

**Keywords:** Pedestrian, Distraction, Cellular phone, Pedestrian distraction, Street crossing

## Abstract

**Background:**

Pedestrians distracted by digital devices or other activities are at a higher risk of injury as they cross streets. We sought to describe the incidence of pedestrians distracted by digital devices or other activities at two highly traveled urban intersections after the implementation of a pedestrian safety intervention at one of the intersections.

**Methods:**

This was an observational field study of two urban intersections. Two investigators were stationed at each of the four corners of the intersection. Each pair of observers included one “person counter” and one “behavior counter”. The “person counter” tallied every individual who approached that corner from any of the three opposing corners. The “behavior counter” tallied every individual approaching from the three opposing corners who were exhibiting any of the following behaviors: 1) eating, 2) drinking, 3) wearing ear buds/headphones, 4) texting, 5) looking at mobile phone or reading something on mobile phone, or 6) talking on mobile phone. Every 15 min, each pair of observers rotated to the next corner of the same intersection, allowing each pair of observers to complete one 15-min observation at each of the four corners of the intersection. Intersection A had stencils at the curb cuts of each corner alerting pedestrians to put down a digital device while crossing the intersection while intersection B did not.

**Results:**

1362 pedestrians were observed; of those, 19 % were distracted by another activity at both intersections. Of the total, 9 % were using ear buds/headphones; 8 % were using a digital device (talking, texting, or looking down at it); and 2 % were eating or drinking. Inter-observer validity among observers (kappa) was 98 %. Of those that were distracted, 5 % were either using an assistive device (cane, walker, motorized scooter) or walking with a child (either on foot or in stroller). There were no differences in the proportion of pedestrians who were distracted at either intersection, except that more pedestrians were talking on a cell phone while crossing intersection B.

**Conclusions:**

It is unclear to what degree a pedestrian safety messaging campaign is effective in decreasing distraction by digital devices. Further evaluation of the effect of posted warnings about pedestrian distraction on the safety of crossing behaviors is needed.

## Background

Distracted walking is defined as walking while simultaneously engaged in other activities, such as using a mobile phone, listening to portable media players such as an IPod or MP3 while wearing headphones, eating or drinking, and conversing with other people (Richtel 2015). Distracted walkers tend to have reduced awareness of their surroundings (Byington and Schwebel 2013; Hatfield and Murphy 2007; Lamberg and Muratori 2012; Lopresti-Goodman et al. 2012; Nasar and Troyer 2013; Schwebel et al. 2012; Stavrinos et al. 2009; Thompson et al. 2012). More than half of all adult cell phone owners have experienced distracted walking encounters where they have either bumped into a distracted pedestrian or where they have physically bumped into another person or object because they were distracted by using their own cell phone (Smith 2014).

Walking while using a mobile phone can be especially dangerous. In 2010, 1506 pedestrians were evaluated in US emergency departments due to injuries sustained while using a mobile phone (Nasar and Troyer 2013). The majority of those injuries were associated with talking on a mobile phone (69.5 %), but 9.1 % were associated with text messaging (Nasar and Troyer 2013). As texting becomes ubiquitous among mobile phone users, injury prevention advocates must consider the increasing risk for injury among pedestrians. The phenomenon of using a mobile electronic device for texting, talking, or listening to music by a pedestrian has been called “digital distraction”.

Among the nation’s medium-sized cities (populations between 100,000 and 199,999), New Haven ranks fifth with 12 % of its citizens who walk to work on a daily basis (Connecticut-by-the-numbers.com 2014). In Spring of 2008, two young pedestrians were struck and killed by cars. These tragedies occurred within a short period of time of each other and involved a promising, young medical student and an 11-year-old girl. Shortly after these tragedies, the City of New Haven launched a “Street Smarts” educational community campaign that was conceived through a collaboration between the City of New Haven’s Transportation Traffic and Parking Department, Yale-New Haven Hospital, Yale School of Medicine, community groups and city residents. After several months of meeting and planning, in the Fall of 2008, *Street Smarts*, an education plan that focuses on three critical components was unveiled as follows: SMART Driver; highlighted driver responsibilities and a call for patience, civility, and increased observation: SMART Cyclist; educating the rights and responsibilities of cyclists, while listing best practices and promotion of cycling throughout the city: and finally, SMART Walker; to promote walkability with a particular focus on school-age youth. The message of this community campaign was that Street Smarts go beyond simply obeying the traffic regulations or driving below the speed limit; all users of the streets must be attentive at all times and be patient and willing to share the roadways with other users (City of New Haven 2014).

**Fig. 1:**
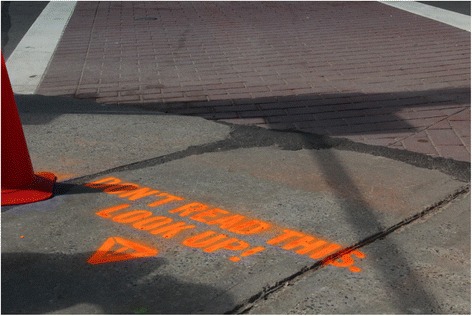
Sidewalk stencil at intersection A

Additionally, the City of New Haven passed the Complete Streets Legislation in 2009 that developed a complete street policy that included developing a design manual and community planning process, and working with the New Haven Police department to develop traffic safety benchmarks to improve the safety and visibility of pedestrians, cyclists, and transit users on the city’s streets (Lynch 2008). This policy has allowed for safety improvements at several intersections where pedestrian fatalities have occurred such as timing of the vehicular and pedestrian traffic signals, high visibility crosswalk striping, signage alerting vehicles of an upcoming pedestrian crossing, and sidewalk stenciling to alert pedestrians to avoid distraction as they cross intersections (Fig. 1) (Giraldo and Ligata 2013). This educational campaign based on sidewalk stenciling is similar to that done in other cities such as New York (Schweber 2012). The efficacy, however, of such campaigns to reduce the incidence of distracted pedestrian behavior has not been previously assessed.

Beginning in 2003, the City of New Haven began performing annual point-in-time survey counts of bicycle and pedestrian activity at key intersections. In 2011, the city expanded their focus and interest in the pedestrian and bicyclist counts by adding six new intersections to monitor in more detail the pedestrian and bicyclist traffic in the downtown area. In 2012, the city further expanded the study area by conducting pedestrian and bicycle counts at 15 additional intersections which make the total surveying occurring at 25 intersections for the 2012 study. Counts of pedestrian activity showed that of the four original city intersections counted in previous studies, there was an observed overall decrease of pedestrian traffic (approximately −6 %) from 2011 to 2012 during the midday. However, two of the four major intersections observed experienced pedestrian volumes of over 1000 pedestrians per hour and an overall 14 % increase in bicyclists from 2010. Moreover, pedestrian activity near Yale University and the Medical district decreased slightly (approximately −4 %) from 2011 to 2012 during the midday. The bicycle and pedestrian counts indicated that non-motorized transportation continues to play an increasingly important role in the downtown transportation system (MacBroom and Inc 2012).

The sites chosen for this study were intersections based on the city’s prior point-in-time surveys as well as their high-volume pedestrian, cyclist, and auto traffic with intersection A located in the Medical District and intersection B located in the downtown area deeming them a prime location to conduct this study.

### Review of literature and search strategies

Using Web of Knowledge, PubMed, Social Science Citation Index, Scopus, and Academic Search Premier, a word search was performed with combinations of the phrases “pedestrian”, “walking”, “cellular phone”, “mobile phone”, “injury”, “text”, and “text messaging” and yielded seven articles that addressed pedestrian distraction due to text messaging or internet usage (Byington and Schwebel 2013; Lamberg and Muratori 2012; Lopresti-Goodman et al. 2012; Nasar and Troyer 2013; Schwebel et al. 2012; Thompson et al. 2012; Neider et al. 2010). A separate search of Web of Knowledge, PubMed, Social Science Citation Index, Scopus, and Academic Search Premier using combinations of the phrases “distracted walking”, “cellular phone”, “mobile phone”, “pedestrian”, “pedestrian behavior”, “distraction”, and “safety” resulted in 15 articles relevant to pedestrian distraction due to mobile phone use in general (Byington and Schwebel 2013; Hatfield and Murphy 2007; Lamberg and Muratori 2012; Lopresti-Goodman et al. 2012; Nasar and Troyer 2013; Schwebel et al. 2012; Stavrinos et al. 2009; Thompson et al. 2012; Neider et al. 2010; Brumfield and Pulugurtha 2011; Bungum et al. 2005; Cooper et al. 2013; Nasar et al. 2008; Neider et al. 2011; Stavrinos et al. 2011).

One observational study of 1102 pedestrians crossing high-risk intersections found that 7.3 % were texting and 6.2 % were talking on the phone (Thompson et al. 2012). Talking on a mobile phone while walking is identified as a cognitive distraction that leads to reduced awareness of surroundings (Byington and Schwebel 2013; Lamberg and Muratori 2012). Pedestrians who text are especially at risk for injury because texting causes both cognitive *and* visual distraction (Byington and Schwebel 2013). Street-crossing poses particular danger to texting pedestrians, as they are not focused on the vehicular environment, and are almost four times more likely to engage in unsafe street-crossing behavior (Thompson et al. 2012).

Existing evidence from both simulated and real-world pedestrian environments indicates that texting pedestrians, compared to non-distracted pedestrians, are more likely to engage in several specific unsafe behaviors. Texting pedestrians take longer to cross a street (Thompson et al. 2012), walk a set distance (Lamberg and Muratori 2012; Lopresti-Goodman et al. 2012), less frequently look both ways before crossing (Thompson et al. 2012), are more likely to be hit by a vehicle (Schwebel et al. 2012), more often look away from the street environment while waiting to cross (Schwebel et al. 2012), and are more likely to display reckless maneuvers such as darting in front of traffic (MacBroom and Inc 2012). Pedestrians who used the Internet on their mobile devices while crossing the street in a simulated environment also exhibit similarly dangerous street-crossing behaviors, such as missing opportunities to safely cross, waiting longer than necessary when a safe opportunity to cross arose, spending more time looking away from the road while crossing, and taking longer to cross the street (Byington and Schwebel 2013). These same pedestrians were also more likely to be struck or almost stuck by an oncoming virtual vehicle (Byington and Schwebel 2013).

The gait of pedestrians who perform the task of texting is distinctly different from both non-distracted pedestrians and pedestrians talking on mobile phones (Lamberg and Muratori 2012). Pedestrians who texted while walking toward a previously identified target showed greater lateral deviation from the remembered target, walked longer distances, and took longer to arrive than either their non-distracted or talking counterparts (Lamberg and Muratori 2012). This observational study and another from 2010 conducted by Hyman et al. 2010, confirmed a lack of situational awareness or inattentional blindness. The researchers found that cell phone users walked more slowly, changed directions more frequently, were less likely to acknowledge other people, including a clown on a unicycle in their route.

Additionally, people who walk and text are more likely to unknowingly compensate for their distraction by moving more cautiously about the spaces through which they easily fit (e.g., rotating their bodies excessively to pass through an already adequately wide doorway) (Lopresti-Goodman et al. 2012). However, despite this over-cautiousness, texting pedestrians were still more likely to bump into a doorframe than their non-texting counterparts (Lopresti-Goodman et al. 2012).

There is clear evidence that pedestrians using mobile phones display unsafe street-crossing behaviors and poor navigation (Lopresti-Goodman et al. 2012; Nasar and Troyer 2013; Nasar et al. 2008; Hyman et al. 2010). This study was conducted to evaluate the effectiveness of a city-wide, grassroots pedestrian safety campaign. A secondary aim was to highlight digitally distracted behaviors of pedestrians in two high traffic volume signalized intersections in New Haven, CT where prior point-in-time surveys have been conducted and known fatalities have occurred. The purpose of this study was to determine the incidence of distracted pedestrian behavior at two intersections, one of which had sidewalk stencils that had been installed during the previous 6 weeks and the other did not. This information would serve as a baseline for modification of the content and dissemination of future iterations of this pedestrian educational campaign.

## Methods

Pedestrian volume and distracted behaviors were observed at two intersections located in downtown New Haven, CT. Both intersections had undergone changes in the timing of the pedestrian crosswalk signals to allow more time for pedestrians to cross from corner to corner. These intersections were selected because of known fatalities as well as high-volume auto, pedestrian, and cyclist traffic. Intersection A had been stenciled 6 weeks prior to the observational study and intersection B had not. Intersection A, near a level I trauma hospital, was observed on a weekday from 7:30 am to 8:30 am in early July 2013. Another intersection (intersection B) near a busy downtown bus stop was observed from 10:30 am to 11:30 am that same day. Eight advance-practice nursing students were trained as observers and completed data collection sheets.

The content for these stencils was determined by focus groups that volunteered to provide their opinions as to which stencils would most likely attract their attention and change their behavior (i.e., not be distracted by an electronic device or other activities) when crossing an intersection. These focus groups consisted of Yale University students, staff, and faculty in addition to New Haven residents. Participants ranked the content of each stencil on a Likert scale of 1–10 as to the likelihood they would pay attention to the stencil if they saw it on the sidewalk when they were walking and looking down (1 = not likely; 10 = highly likely). The stencil that had the highest average score was installed at intersection A 6 weeks before the observations took place (Fig. 1).

At each intersection, one pair of observers was stationed on each corner. Each pair of observers included one “person counter” and one “behavior counter”. The “person counter” tallied every individual who approached that corner from any of the three opposing corners. The “behavior counter” tallied every individual approaching from the three opposing corners who were exhibiting any of the following six distracted behaviors: 1) eating, 2) drinking, 3) wearing headphones or ear buds, 4) texting, 5) looking at a mobile phone or reading something on mobile phone, or 6) talking on mobile phone. Every 15 min, each pair of observers rotated to the next corner of the same intersection, allowing each pair of observers to complete one 15-min observation at each of the four corners of the intersection. Each observer also noted the approximate age (adult, child) and race (white-non Hispanic, white-Hispanic, black, Asian) of each pedestrian observed and whether an assistive device (e.g., walker, cane, wheelchair, motorized scooter) was used.

Prior to the study, inter-rater reliability among observers (kappa) was established by both instructors evaluating their own scoring of behaviors followed by the students completing several “dry runs” on scoring behaviors. A 98 % inter-observer validity accuracy rate was found in scoring behaviors and estimating race and age and assistive device use. To ensure continued accuracy, each instructor remained on separate corners throughout the data collection to ensure that inter-rater reliability was maintained. In addition, the instructors instituted a brief “huddle” after each 15-min rotation and completed a comprehensive debriefing at the end of each site location to gather and categorize any problems identified and resolve any outstanding concerns the data collectors may have had.

For categorical data, the Pearson chi-square test (with Yates correction as necessary) was used to compare demographic and behavioral characteristics of the pedestrians observed at intersections A and B, the positive and negative screen groups. IBM SPSS Statistics Version 22 was used for statistical analysis. The Human Research Protection Program of Yale School of Medicine approved this study.

## Results

For the two intersections, the behavior of 1362 (total) pedestrians was observed: 655 pedestrians at intersection A and 707 pedestrians at intersection B. Demographic characteristics were significantly different between the two intersections with respect to the race of pedestrians and the use of assistive devices (e.g., canes, walkers, wheelchairs). At intersection A, we observed 50 % White (*n* = 327), 15 % Black (*n* = 98), 10 % Hispanic (*n* = 66), and Asian 25 % (*n* = 25 %) compared to intersection B with 30 % White (*n* = 212), 33 % Black (*n* = 233), 33 % Hispanic (*n* = 233), and 2 % Asian (*n* = 14) (Table 1). In addition, at intersection A, we observed 0 % individuals using assistive devices and four (0.6 %) individuals walking with children as compared to intersection B with six (0.8 %) using assistive devices and seven (10 %) walking with children.

**Table 1:** Intersection characteristics

	Intersection A	Intersection B	*p* value
	*n* = 655	*n* = 707	
White	327 (50 %)	212 (30 %)	<0.0001
Black	98 (15 %)	233 (33 %)	<0.0001
Hispanic	66 (10 %)	233 (33 %)	<0.0001
Asian	164, (25 %)	14 (2 %)	<0.0001
Assistive Devices	0 (0 %)	6 (0.8 %)	0.032
Walking with children	4 (0.6 %)	7 (10 %)	0.434

Pedestrians were observed to be using headphones/ear buds (*n* = 119; 9 %), using a digital device (talking, texting or looking down at it) (*n* = 109; 8 %), or eating or drinking (*n* = 38; 2 %) while crossing the intersection (Table 2). There was no difference in the total proportion of pedestrians observed to be distracted at either intersection (*p* = 0.728). At intersection B, however, significantly more pedestrians were talking on a cell phone (4.5 % versus 2 %; X2 = 0.090; *p* = 0.009; OR 0.437; 95 % CI 0.232–0.828). It was also noted that all pedestrians who were pushing a stroller put the stroller down from the curb into the intersection before the walk signal flashed and that two pedestrians using walkers stopped in the middle of the intersections and were still walking and using a mobile electronic device when traffic started moving into the intersection.

**Table 2:** Proportion of pedestrians observed with distracted behaviors

Proportion	Intersection A	Intersection B	Odds ratio	95 % CI
	*n* = 655	*n* = 707		
	*n* (%)	*n* (%)		
Distracted	118 (0.18 %)	134 (0.19 %)	0.866	0.742–1.112
Eating	5 (0.70 %)	4 (0.57 %)	1.352	0.364–5.00
Drinking	12 (1.80 %)	17 (2.40 %)	0.897	0.433–1.867
Texting	18 (2.7 %)	15 (2.10 %)	1.304	0.658–2.549
Talking	13 (2.0 %)	32 (4.50 %)	0.427	0.232–0.828
Ear buds	59 (9.0 %)	60 (8.50 %)	1.033	0.709–1.50

## Discussion

While prior distracted walking research has identified behaviors such as eating, listening to media players, walking and talking with other people, and talking on mobile phones (Thompson et al. 2012), very little research has focused specifically on “digitally distracted” pedestrians who text while walking. Our pilot project sought to capture rates of digital distraction and other distracted walking behaviors as well as the effect of a pedestrian educational intervention that targets distracted walking.

We did find some differences in the rates of distracted behaviors between the two intersections, with fewer pedestrians talking on a cell phone while crossing intersection A that had stenciled warnings about walking while looking down at a device. Whether this difference is due to the effect of the stenciled warning itself or due to demographic differences in the races of pedestrians at each intersection is not clear. Because no observational data is available from the time period before the stencil warning was posted, no conclusions can be drawn from these findings.

We also observed the use of assistive devices among pedestrians, including canes, walkers, and wheelchairs; crosswalk signals were rarely long enough to allow these pedestrians to safely cross the street before vehicle traffic again had the right of way. While digital distraction among pedestrians using assistive devices was not a focus of this investigation, this is a behavior that should be further investigated.

While this study may be similar to other research in that it looks at behaviors in crosswalk there are aspects that are unique. To date, this is the first evaluation of the effectiveness of stenciled sidewalk warnings on the incidence of distracted pedestrian behaviors at an intersection. The method by which we determined the proportion of pedestrians who were distracted has not been described elsewhere. It was found to have excellent agreement among observers with respect to counting the number of pedestrians who were distracted and will serve as the basis for any future study of pedestrian distraction and intersection-crossing behaviors.

This study had several limitations. First, only two busy intersections were observed on a single day; future work may include more busy intersections observed for longer periods of time. The observation times were not the same due to the fact that the same research personnel were used to observe each of the two sites. Second, as we were interested in establishing rates of digital distraction among distracted walkers in downtown New Haven, we found racial differences in the proportions of pedestrians between the two intersections and did not perform observations of more complex behaviors, such as distracted walkers crossing an intersection diagonally, distracted walkers using assistive devices, and distracted walkers crossing against the walk signal. Some of the demographic differences may be explained by the time of day of data collection, especially when one period is during peak commute hours. If intersections are paired for comparison, then different pairs could have different data collection times to identify whether time of day has an effect on behavior. These outcomes will serve as the basis for upcoming studies and will inform future decisions about the content of forthcoming iterations of the Street Smarts Campaign.

## Conclusions

It is unclear what impact of a pedestrian safety messaging campaign can have in decreasing pedestrians distracted by digital devices while crossing an intersection. Following two pedestrian fatalities, the City of New Haven, CT implemented a “Streets Smarts” educational community campaign that focuses on the following three critical components: SMART Driver, SMART Cyclist, and SMART Walker and includes sidewalk stenciling to alert pedestrians to avoid distraction as they cross intersections. Pedestrians at two intersections (one with stenciling and one without) were observed for distracted behaviors. Fewer pedestrians were observed talking on a cell phone while crossing intersection A that had stenciled warnings about pedestrian distraction than intersection B that did not have the stenciling warnings. Further evaluation of the effect of posted warnings about pedestrian distraction on the safety of crossing behaviors is needed.
